# Commentary: Arnica Montana Effects on Gene Expression in a Human Macrophage Cell Line: Evaluation by Quantitative Real-Time PCR

**DOI:** 10.3389/fimmu.2016.00280

**Published:** 2016-09-08

**Authors:** Salvatore Chirumbolo, Geir Bjørklund

**Affiliations:** ^1^Unit of Geriatry, Department of Medicine, University of Verona, Verona, Italy; ^2^Council for Nutritional and Environmental Medicine, Mo i Rana, Norway

**Keywords:** *Arnica*, homeopathy, gene expression profiling, inflammation, sesquiterpenes

Olioso et al. described the effect of *Arnica montana* L on the gene expression of the human monocytic leukemia cell line THP-1, reporting that homeopathic preparations of *Arnica* upregulated the expression of genes, such as CCL7, CXCL1, CXCL2, interleukin 8, and bone morphogenetic protein 2 (BMP2) (1). Plants from the genus *Arnica* are sources of anti-inflammatory compounds and homeopathic formulations were reported to be effective in treating inflammatory ailments (2–4), despite some negative or controversial evidence (5, 6). Olioso et al. showed effects of *Arnica* homeopathic preparations from 2c to 15c, on the gene expression of several cytokines and chemokines, involved in the recruitment and activation of neutrophils and other innate immune cells using a qRT-PCR (1). Bioactive components from the disk flowers of *Arnica* species are sesquiterpene lactones, e.g., chamissonolid, helenalin, and 11α,13-dihydrohelenalin (7–9). The concentration of these molecules in *Arnica* homeopathic preparations was not chemically defined, apart from the evaluation as 0.036% sesquiterpene lactones in the raw alcoholic extract (1). Usually, the amount of total helenalin and total 11α,13-dihydrohelenalin is 5.6%, i.e., 1.1 mg and 5.1%, i.e., 1.0 mg, respectively (10). This would mean that in the starting 30% alcoholic preparation of *Arnica* (1c), a percentage of 0.036% sesquiterpene lactones, should correspond to 0.72 μg, i.e., 72 pg in the dilution 2c (1), quite far from the activity range reported elsewhere (9, 10). Surprisingly, Olioso et al. reported an activity on NF-κB gene expression in any dilution used (except for 15c) only in cells treated 24 h and activated with IL-4. Aside for the upregulation of *rela-*A gene at 2 h, Olioso et al. did not show any effect of *Arnica* preparation 2c, despite the existence of further reports published elsewhere showing an effect of sesquiterpene lactones on NF-κB (11, 12). The authors did not report reproducible data for the highest concentrated dilution used in their study, i.e., *Arnica* 2c, which appeared to exhibit a significant action quite only on THP-1 cell line following activation with IL-4 [Table 3B in Ref. (1)]. They did not ascertain the absence of outlying observations in the experimental data. It is mandatory that, before calculating the central tendency and dispersion parameters based on the outlier-based indicators (e.g., mean, SD, SEM), the data should be subjected to the discordancy tests (13–16). Showing only SEM or other parameters excluding raw data cannot allow the reader to realize about any statistical reliability and reproducibility. More importantly, the 95% confidence limits of the mean values [Table 2A in Ref. (1)] are not distinguishable from zero implying no effect.

We evaluated statistic distribution of SEM variability to ascertain if any reported outliers due to bias error affected the parametric or non-parametric behavior of the investigated samples. The authors used Wilcoxon test, although no special requirements to call for a non-parametric test were requested (17). Effects reported as changes *p* < 0.05 in the gene expression pattern amounted to 4 and 7 (2 and 24 h, respectively, M0) and to 8 and 12 (2 and 24 h, respectively) for THP-1 monocytes treated with IL-4 (M2-type), where *p* < 0.01 where 13 (M2) vs. 4 (M0) (1). Probably, cells undergoing less stress (24 h incubation at 37°C 5% CO_2_) and addressed to drive any molecular machinery to a highly controlled response to a stimulus (i.e., IL-4) amplified signals vs. noises, i.e., reduced the difference between noises and statistical variability.

A statistical analysis of the reported data in Tables 2A, 2B, 3A, and 3b of Ref. (1) showed that intra-assay and inter-assay variability, expressed as SEM, contained possible bias (heterogeneous variance) due to the dispersion effect of the reported variability and outlying data. In this context, SEMs appeared to have been log-normally distributed, as evaluated in a goodness-of-fit test, with some exception, particularly in experiments where THP-1 followed the IL-4 treatment (Cramer–von Mises test: 2c, *p* = 0.676; 3c, *p* = 0.279: 5c, *p* = 0.254; 9c, *p* = 0.792. Data were confirmed in a Kolmogorov–Smirnov test and the Shapiro–Wilk test, and only the D’Agostino–Pearson test gave 15c, *p* = 0.07) (18). A possible carry over effect on SEM was caused by data containing outliers. Only one or two outlying observations were identified, especially by the more powerful Grubbs, skewness, and kurtosis discordancy tests (Dixon tests generally failed) (18). A Rosner’s extreme studentized deviate test for multiple outliers (two-sided test, *z* = 3.5), showed, respectively 18 and 14 outliers in the SEM distribution of M0-type TPH-1 cell line and 4 and 0 for SEM distribution in M2-type THP-1 cell line, confirming a more constant dynamics of gene expression in M2-cells (1, 19). Outliers in the SEM columns probably biased any statistic significance, as variability in the elaborated data was heterogeneously dispersed. The Dixon’s Q-test for single outlier detection showed that the most of outliers occurred in the evaluation of CCL7 gene expression in the M0 experimental group (2 h: 3c, *p* < 0.002; 9c, *p* = 0.004; 15c, *p* = 0.022; 24 h: 2c, *p* = 0.003; 3c, *p* = 0.002; 5c, *p* = 0.002; 15c, *p* = 0.002), while in the M2 group they occurred only in the 2 h cluster (5c CCL17, *p* < 0.002; 9c CCL2, CCL5, *p* < 0.05; 15c CCL7, *p* < 0.04) (1), results confirmed with Grubbs’ test (20). Although the authors showed skewness (1), variance heterogeneity might affect the decision about which test should adopt.

Figure 1 reports that this difference, calculated between each gene for each dilution, appeared significant only in the 2 h group (2c, *p* = 0.000865; 5c, *p* = 0.0134, 15c, *p* = 0.00020) but also that regression data for the other comparisons contained some bias, regarding either normal fit, Durbin–Watson test or the constant variance test (CVT) (1, 19). The presence of discordant outliers in bivariate data should enforce our conclusion (13, 21, 22).

**Figure 1:**
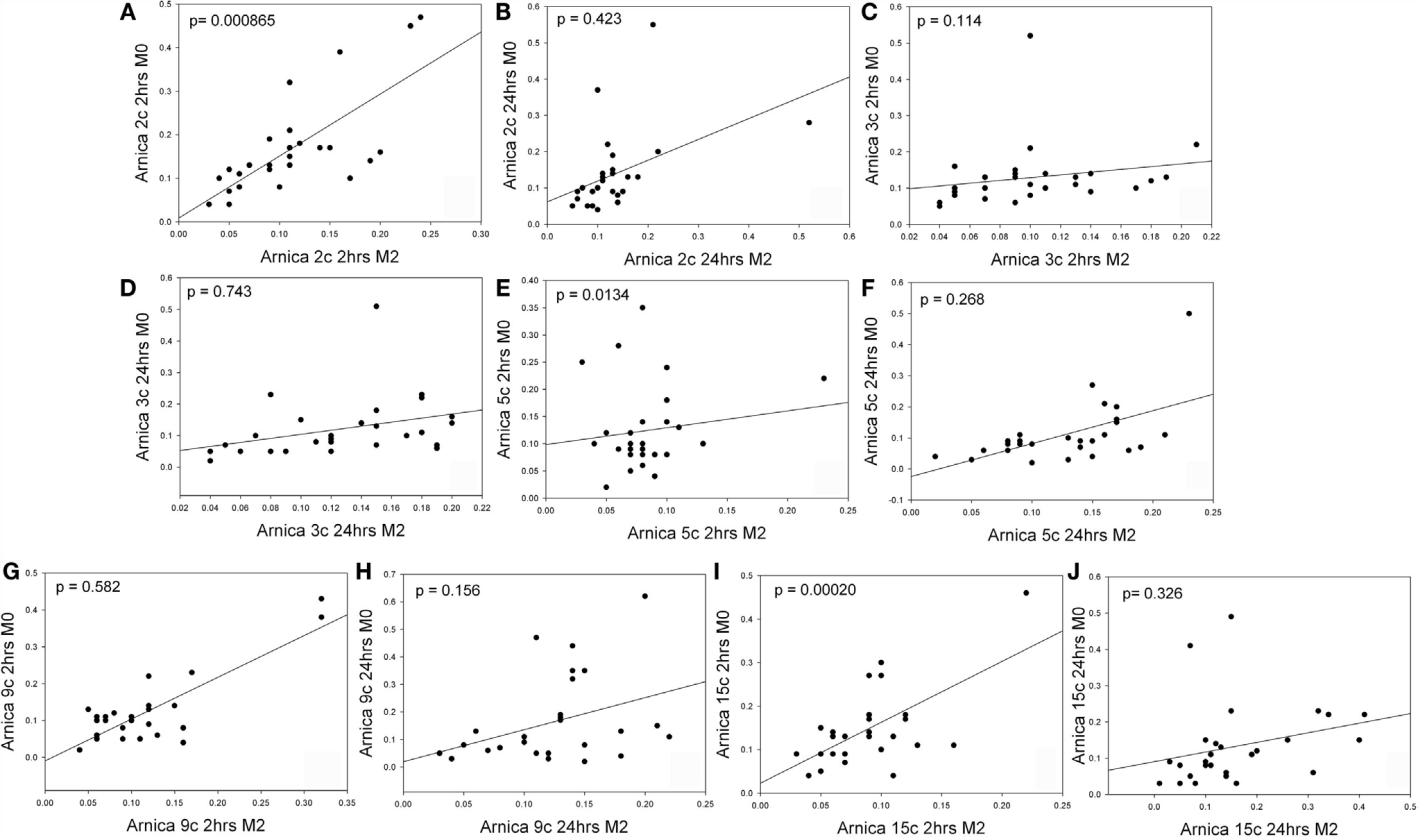
**Linear regression of non-original data [reported from Ref. (1) in the text] elaborated by evaluating SEM distributions of Tables 2A, 2B, 3A, and 3B of Ref. (1)**. Comparisons were made with a Wilcoxon–Mann–Whitney U-test or an ANOVA and paired *t*-test depending on the log-normal distribution. Panels data are described as follows (tests are passed unless they are not indicated). **(A)** *r* = 0.7239, *p* < 0.0001, DW test = 1.9546, KW test = 0.7393, *CVT failed, p* = 0.0071; **(B)** *r* = 0.4521 *p* = 0.0157, DW = 1.9729, *KS failed, p* = 0.0115; CVT, *p* = 0.1510; **(C)** *r* = 0.2022, *p* = 0.3022, DW = 1.8110, *KS failed, p* = 0.0484; CVT, *p* = 0.7603; **(D)** *r* = 0.3367, *p* = 0.0798, *DW failed* = 1.4179, KS, p = 0.1025; CVT, *p* = 0.2199; **(E)** *r* = 0.1440, *p* = 0.4647, DW = 2.0089, KS, *p* = 0.1900; CVT, *p* = 0.8204; **(F)** *r* = 0.5721, *p* = 0.0015, *DW failed* = 1.1683, KW, *p* = 0.2954; *CVT failed, p* = 0.0021; **(G)** *r* = 0.8208, *p* < 0.0001, DW = 1.9817, KS, *p* = 0.9823; CVT, *p* = 0.1621; **(H)** *r* = 0.3741, *p* = 0.0498, DW = 1.5226, KS, *p* = 0.1999; *CVT failed, p* < 0.0001; **(I)** *r* = 0.6279 *p* = 0.0003, DW = 2.0124, KS, *p* = 0.6183; *CVT failed, p* = 0.0214; **(J)** *r* = 0.2657, *p* = 0.1717, *DW failed* = 1.2594, KS, *p* = 0.1417; CVT, *p* = 0.9967.

Statistic significance appeared as highly frequent in a more stable experimental condition and in dilutions with active principles very far from a pharmacological bioactivity and without an apparent dose–response behavior. Probably, not all the distributions should be evaluated with a Wilcoxon due to the existence of outliers much more frequent only in certain experimental conditions.

In conclusion, data arrays as presented by the authors may contain some discordant outlying observations, thus affecting results reliability. The effects of *Arnica* water/ethanol extracts should be, therefore, replicated.

Our consideration is that by replicating the data, we could appreciate if homeopathic *Arnica* is really effective on inflammatory gene upregulation in innate immune cells.

## Author Contributions

SC read the original paper, discussed it with GB, statistics were re-evaluated by SC with GB, GB gave insights from the literature, SC re-wrote the paper, GB revised it, and SC reviewed the revision and submitted the paper once GB has given his agreement.

## Conflict of Interest Statement

The authors declare that the research was conducted in the absence of any commercial or financial relationships that could be construed as a potential conflict of interest.
